# Percutaneous uniaxial endoscopic fenestration for removal of symptomatic cervical Tarlov cyst

**DOI:** 10.1186/s12891-026-09954-z

**Published:** 2026-05-21

**Authors:** Bin Tang, Libin Li, Yuanming Zhong, Yongqi Chen, Baohua Huang

**Affiliations:** 1 The First Affiliated Hospital of Guangxi University of Chinese Medicine, No.89-9, Dongge Road, Qingxiu District, Nanning, 530023 Guangxi Zhuang Autonomous Region P.R. China; 2 Guangxi University of Chinese Medicine, Nanning, Guangxi Zhuang Autonomous Region P.R. China

**Keywords:** Percutaneous uniaxial endoscopy, Cervical keyhole, Tarlov cyst, Perineural cyst

## Abstract

**Background:**

To report a rare case of cervical Tarlov cyst successfully treated using a percutaneous uniaxial endoscopic fenestration.

**Methods:**

This was a single case report describing the surgical technique, clinical assessment, radiological verification, and long-term follow-up outcomes of percutaneous uniaxial endoscopic surgery for symptomatic cervical Tarlov cyst.

**Clinical presentation:**

A 43-year-old male patient presented with intermittent pain and numbness in his right forearm for 2 years, with exacerbation over the past month. The symptoms were aggravated by fatigue and upon waking in the morning, and conservative treatment yielded no effect. MRI revealed a C8 Tarlov cyst. The patient underwent cervical percutaneous endoscopic laminar decompression and Tarlov cyst resection. Cervical MRI performed on the 2nd postoperative day confirmed complete cyst resection. Postoperatively, the patient’s radicular pain almost completely resolved without symptoms of cerebrospinal fluid leakage, and no recurrence of pain was reported during a 2-year follow-up.

**Conclusion:**

This case report describes the safe and successful application of percutaneous uniaxial endoscopic fenestration combined with cervical Tarlov cyst resection.

## Background

Tarlov cyst, also known as perineural cyst or nerve root sheath cyst, is formed by the accumulation of cerebrospinal fluid between the endoneurium and perineurium of spinal nerve roots [1]. Most scholars consider it a congenital or spontaneous dural diverticulum or arachnoid hernia, resulting from a congenital defect of the dura mater. Essentially, it is a non-neoplastic spinal arachnoid diverticulum. The cyst originates between the endoneurium and perineurium of the anterior or posterior roots of spinal nerves, either surrounding and invading nerve fibers or being enclosed by them [2].

The global pooled prevalence of Tarlov cysts was 4.18%, with a higher prevalence in females [3]. The cysts are located around the spinal nerve roots within the spinal canal; the vast majority occur in the lumbosacral segment of the spinal cord, and only a very small number occur in the cervical and thoracic vertebrae [4]. Cervical Tarlov cysts can cause neck and shoulder pain, as well as sensory abnormalities such as upper limb pain, burning sensation, and numbness, which are consistent with the nerve innervation area. Symptoms are caused by compression, traction, or torsion of the cyst-bearing nerve root and/or surrounding nerve roots [5].

### Clinical presentation

A 43-year-old male patient presented with pain and numbness in the right forearm for 2 years, which worsened over the past month. He complained of intermittent numbness and pain in the right elbow and forearm, particularly aggravated by fatigue and in the early morning. There was no pain or numbness in the left upper limb, nor weakness in both upper limbs. Conservative treatment was previously administered, but the pain symptoms showed no significant improvement. The patient had a history of " hepatitis B with triple positive serology” and was currently receiving regular antiviral treatment with tenofovir alafenamide fumarate tablets. No other relevant medical history was noted.

Physical examination revealed extensive tenderness in the right neck and shoulder muscles. The right brachial plexus traction test was positive, and the right intervertebral foramen compression test was positive. The right elbow Tinel sign was negative, and bilateral Hoffman signs were negative. The skin sensation, muscle strength, and muscle tone of both upper limbs were normal. The biceps reflex, triceps reflex, and radial periosteal reflex were all normal, and no pathological reflexes were elicited in the lower limbs. The Visual Analog Scale (VAS) score for upper limb pain was 6 (range: 0–10), and the Neck Disability Index (NDI) was 15% (range: 0%–100%).

**Fig. 1:**
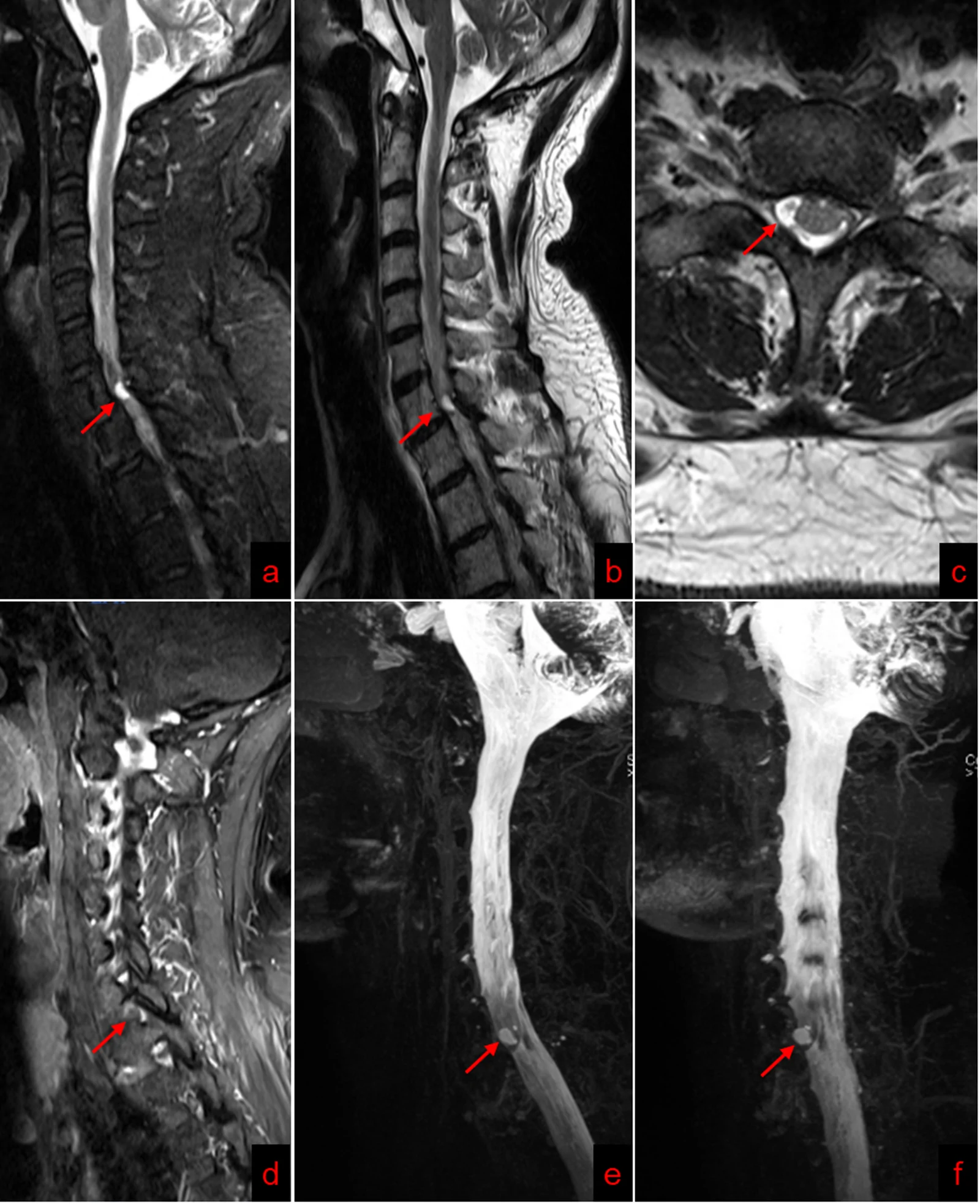
Preoperative MRI noting the C8 Tarlov cyst (red arrow). **a** T2-weighted fat-suppressed imaging. **b**, **c** T2-weighted imaging. **d** Gadolinium-enhanced MRI. **e**, **f** 3D reconstruction of T2-weighted MRI myelography

In imaging examinations, MRI showed a high-signal cyst on T2-weighted imaging, with nerve roots passing through the cyst. No enhancement of the cyst was observed on contrast-enhanced MRI. MRI myelography revealed a distinct hyperintense cyst involving the right C8 nerve root, suggesting that the cyst might communicate with the subarachnoid space and be filled with cerebrospinal fluid (Fig. 1).

The treatment plan involved spinal endoscopic laminotomy for decompression, resection of the Tarlov cystic space-occupying lesion, and nerve root decompression performed under general anesthesia.

**Fig. 2:**
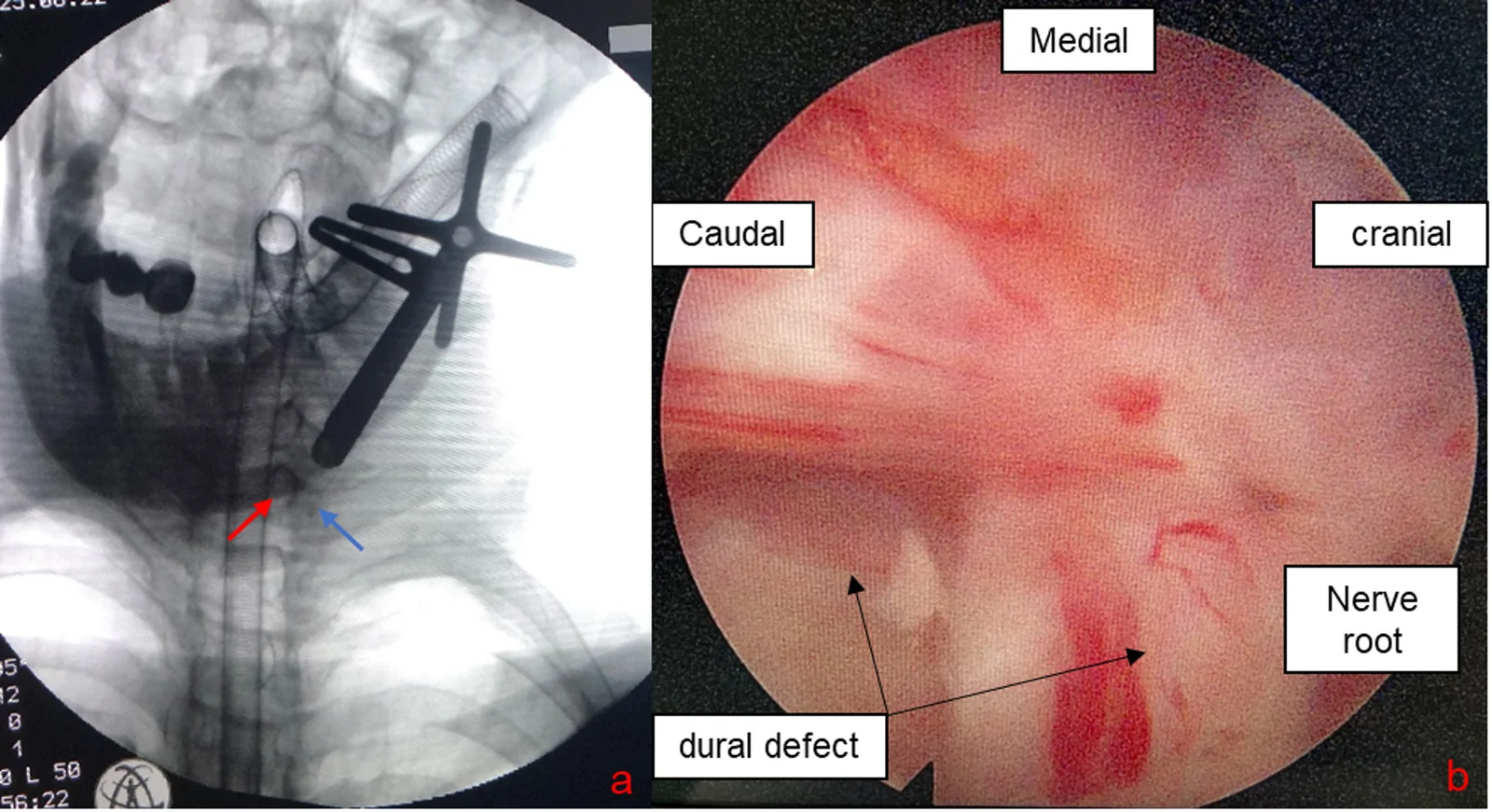
**a** Anteroposterior view reveals the working cannula between the spinous process (red arrow) and the medial border of pedicle (blue arrow), which is located at the level of the C7/T1 intervertebral foramina. **b** Endoscopic view after cyst removal

**Fig. 3:**
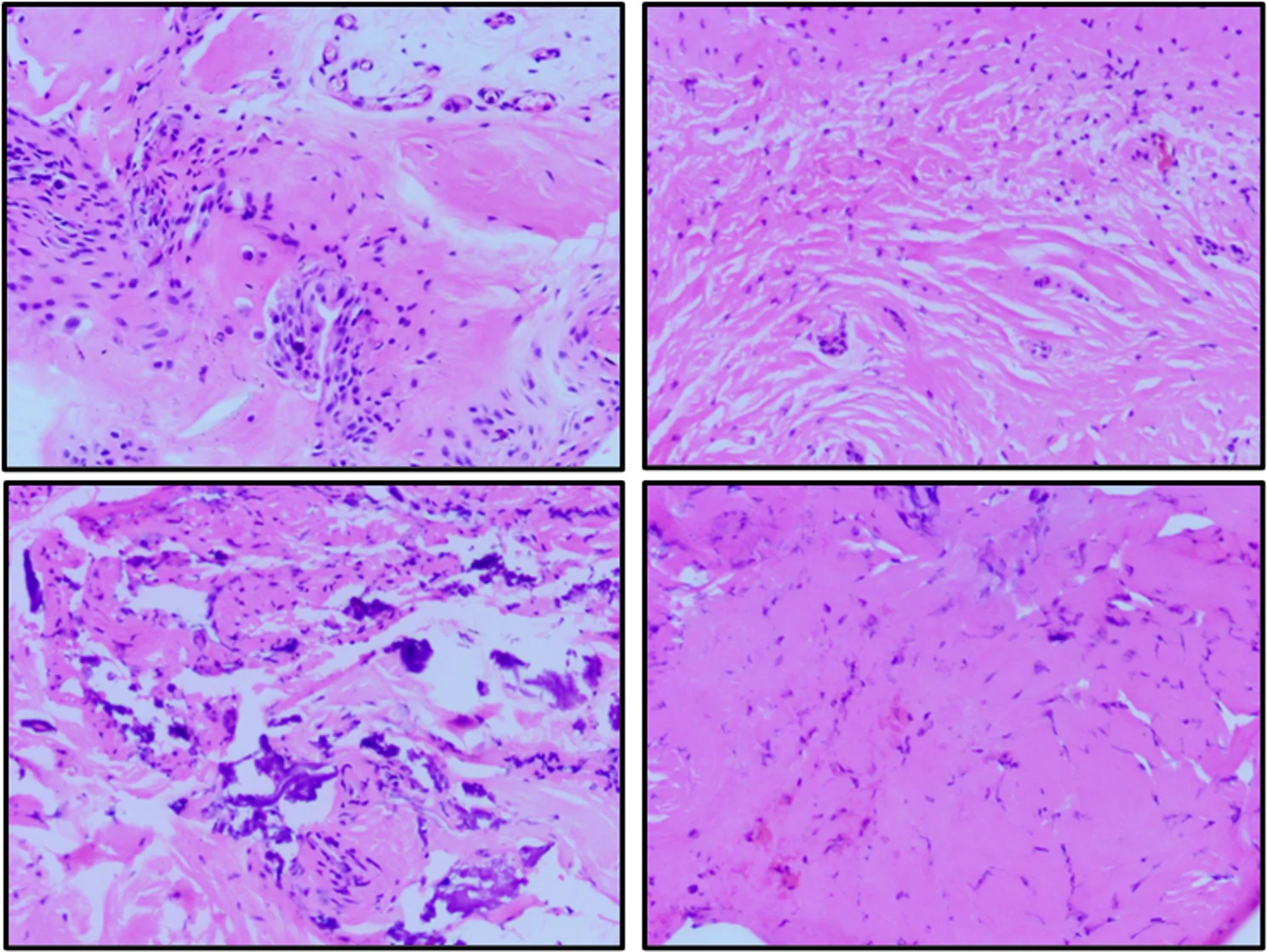
Pathological examination result: Fibrous connective tissue, cartilage, and fragmented bone tissue were identified; no tumor tissue was found. PAS staining showed no abnormalities

The patient was placed in the prone position, and skin marking was performed with a marker pen. The surgical field was routinely disinfected with iodine tincture and alcohol, followed by draping with sterile towels, application of a skin protective film, and placement of medium drapes and small-hole drapes. Fluoroscopy with a C-arm was used to localize the right C7/T1 intervertebral space, and the midline of the spinous process was marked. A puncture point was marked 2 cm lateral to the midline of the C7/T1 intervertebral space. After successful anesthesia, the surgical area was draped under sterile conditions, and a surgical skin protective film was applied. The puncture needle was positioned, and after confirming the puncture site, a guide wire was inserted. A sharp blade was used to make a 0.6 cm incision at the puncture site. Sequentially, 1st to 3rd grade dilating cannulas were screwed in along the guide wire. Under the protection of the sleeve, a trephine was used for bone shaping and anchoring to the outer cortical bone of the lamina (Fig. 2a). C-arm fluoroscopy confirmed that the front end of the cannula was at the C7/T1 intervertebral space in the anteroposterior view and at the posterior edge of the C7 lamina in the lateral view, and a working channel was inserted. Continuous normal saline irrigation was performed, and electrocoagulation hemostasis was achieved with a radiofrequency ablation electrode under endoscopic visualization. A high-speed diamond burr was used for laminotomy and decompression to expose the medial orifice of the C7/T1 intervertebral foramen, as well as the dura mater on the surface of the cervical spinal cord and the C8 nerve root. Endoscopic rongeurs were used to debride the tissues around the exiting nerve root and lateral to the dural sac, and endoscopic nucleus forceps were used to remove the cystic tissue, fully releasing the nerve root and relieving compression. After completion of cyst resection, two dural defects of approximately 2 mm each were identified at the junction of the dorsal side of the exiting cervical nerve root, the lateral side of the cervical dura mater, and the medial side of the nerve root (Fig. 2b). Subsequently, autologous fat around the nerve root was harvested and placed to seal the dural defects. Finally, the endoscopic channel was removed, and the skin incision was sutured. The excised cyst wall tissue was sent for pathological examination. The report indicated the presence of fibrous connective tissue, cartilage, and fragmented bone tissue; no tumor tissue was identified, and PAS staining showed no abnormalities (Fig. 3).

Postoperatively, the patient reported that the preoperative radicular pain was almost completely relieved, with no symptoms related to cerebrospinal fluid leakage. The patient was advised to take oral mecobalamin tablets for neurotrophic support.

Cervical CT and MRI reexaminations were performed on the 2nd postoperative day. Comparison with preoperative imaging revealed no residual perineural cysts. The patient was discharged on the 3rd postoperative day, with a Visual Analog Scale (VAS) score of 0 for upper limb pain and a Neck Disability Index (NDI) of 5%.

**Fig. 4:**
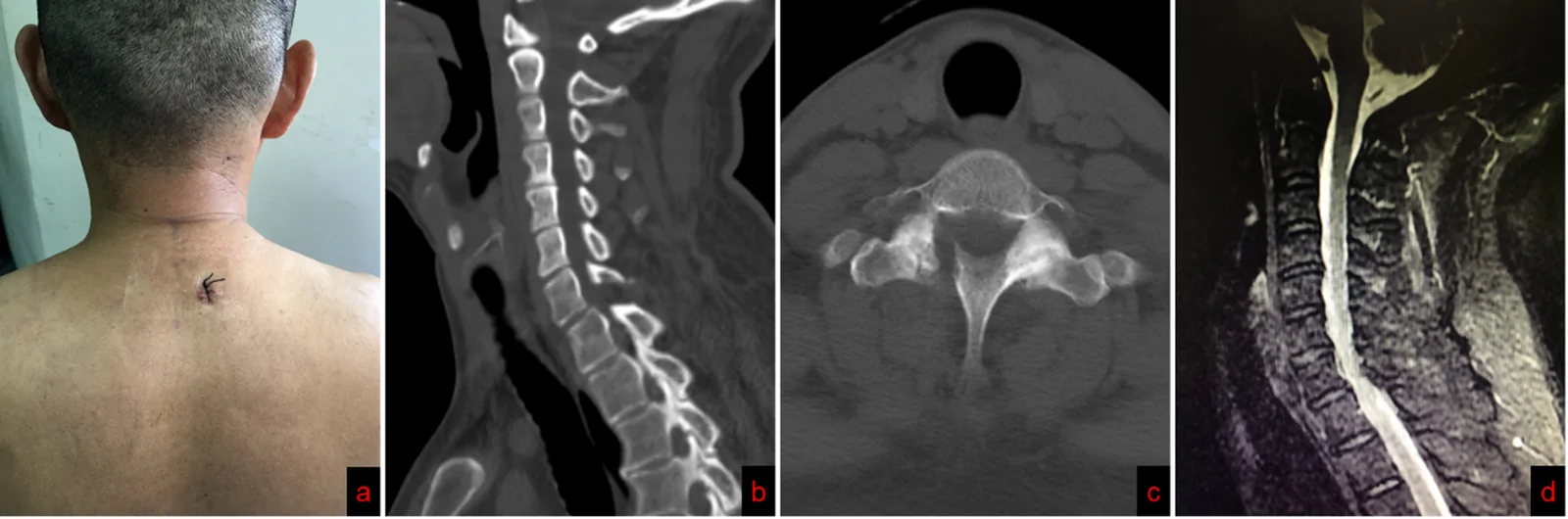
**a** Postoperative incision status. **b**, **c** Postoperative CT scan images. **d** Postoperative MRI scan images

The patient was followed up by telephone for 2 consecutive years, and no symptom recurrence was reported during the entire follow-up period (Fig. 4).

## Discussion

Extramedullary spinal cysts are classified into three categories: meningeal cysts, epidural non-meningeal cysts, and neuroenteric cysts. Meningeal cysts consist of three types: Type Ⅰ (epidural cerebrospinal fluid cysts without nerve fibers), Type Ⅱ (epidural meningeal cysts containing nerve fibers, i.e., Tarlov cysts), and Type Ⅲ (intradural meningeal cysts) [2, 6].

Tarlov cysts (perineural cysts), neurofibromas, and schwannomas are all spinal and peripheral nerve-related lesions. All three may cause pain, numbness, and sensory abnormalities in the corresponding innervated areas, requiring clinical differentiation [7]. Symptoms of Tarlov cysts are mostly related to postural changes and often present intermittently. Neurofibromas and schwannomas manifest as persistent nerve compression symptoms. In terms of pathological nature, Tarlov cysts are benign cystic lesions filled with cerebrospinal fluid, located between the nerve fascicles and endoneurium of spinal nerve roots, without tumor cell components. They are mostly caused by abnormal cerebrospinal fluid circulation, trauma, or inflammation [8]. Neurofibromas are benign tumors originating from neurofibroblasts, which can occur in nerve trunks, nerve roots, or nerve endings. Composed of nerve fibers, fibroblasts, and Schwann cells, they are often associated with neurofibromatosis (e.g., Type NF1). Schwannomas are benign tumors originating from Schwann cells, growing around the nerve sheath. Compared with neurofibromas, they have a simpler cellular composition (predominantly Schwann cells), are mostly solitary lesions, and rarely undergo malignant transformation. On MRI examination, Tarlov cysts have signal intensity consistent with cerebrospinal fluid, showing high signal on T2-weighted imaging and low signal on T1-weighted imaging, with no enhancement on contrast-enhanced scanning. Neurofibromas present mixed high signal on T2-weighted imaging and isointense or low signal on T1-weighted imaging; they have relatively clear borders, may be lobulated, and show mild to moderate heterogeneous enhancement on contrast-enhanced scanning. Often distributed along the nerve course, they can cause thickening of the nerve trunk, and some may undergo cystic degeneration, but the cystic degeneration area does not have cerebrospinal fluid signal characteristics. Schwannomas show high signal on T2-weighted imaging and isointense or low signal on T1-weighted imaging; they have clear borders and regular shapes, mostly round or oval, and some may present a “target sign” (low signal in the center and high signal in the periphery); they show obvious homogeneous enhancement on contrast-enhanced scanning [9]. On CT, Tarlov cysts appear as low-density lesions consistent with cerebrospinal fluid, with no bone destruction or only mild compressive bony changes. Neurofibromas and schwannomas appear as soft tissue density lesions; when large, they can cause erosion of surrounding bones and enlargement of intervertebral foramina [10]. Tarlov cysts can show communication with the subarachnoid space through myelography or MRI myelography; neurofibromas and schwannomas usually do not have this feature on angiography, only showing space-occupying filling defects [11]. Sometimes, Tarlov cysts at the C8–T1 level have similar symptoms to cubital tunnel syndrome [12]. Electromyography of Tarlov cysts shows normal sensory potential and conduction velocity of the ulnar nerve, which, combined with MRI, can achieve good differential diagnosis [13].

Only a small proportion of patients with symptomatic cervical Tarlov cysts require treatment, which includes conservative management and surgical intervention [14]. Conservative treatments consist of wearing a soft cervical collar to restrict movement, transcutaneous electrical nerve stimulation (TENS), manual therapy, rehabilitation involving passive stretching and exercises, active functional training, oral corticosteroids, and intervertebral foramen glucocorticoid injections [15–17]. Open surgical intervention for symptomatic cervical Tarlov cysts can effectively improve patients’ symptoms and quality of life [18]. Meanwhile, there are literature reports on the use of percutaneous uniaxial endoscopic fenestration and percutaneous biportal endoscopic fenestration for the treatment of lumbar or thoracic Tarlov cysts [19–21]; however, there are currently no reports on the treatment of cervical Tarlov cysts using percutaneous uniaxial endoscopy.

During surgery, the clavicle and mandible may cause lateral obstruction during fluoroscopic localization of the cervicothoracic segment. Accurate localization is crucial for the smooth progression of the operation. When performing laminar decompression, caution must be exercised with the powered burr to avoid excessive downward pressure, as unexpected penetration could damage the spinal cord and nerves. During cyst dissection, injury to the nerves and dura mater should be avoided, and the cyst wall must be completely resected to prevent recurrence. Due to the thinness of the cyst wall, it is prone to rupture during manipulation, resulting in the inability to visualize the complete cyst morphology under endoscopy. Nevertheless, combined with the localization of the cyst based on preoperative imaging, this does not hinder the complete resection of the cyst under endoscopy.

With the continuous development of spinal endoscopic surgery, surgeons are now able to effectively treat perineural cysts via percutaneous endoscopic surgery, which offers advantages such as small incisions, rapid recovery, and fewer related complications. Although the treatment of this patient was successful, the generalizability of this case report should not be overestimated, and comparative studies with other non-surgical and surgical methods are needed. In addition, large-scale studies and long-term follow-up are required to further evaluate the efficacy of this surgical procedure.

## Conclusion

We reported a case of a patient with neurological symptoms caused by a cervical Tarlov cyst who had undergone successful percutaneous uniaxial endoscopic laminar bony fenestration decompression and cyst resection. Postoperatively, the patient’s pain symptoms completely resolved. With its high-definition imaging and minimally invasive operation, uniaxial endoscopic technology is expanding its application scope in the treatment of spinal diseases.

## Data Availability

Data generated or analyzed during the study are available from the corresponding author by request.
